# In situ chlorophyll fluorescence kinetics as a tool to quantify effects on photosynthesis in *Euphorbia cyparissias* by a parasitic infection of the rust fungus *Uromyces pisi*

**DOI:** 10.1186/s13104-015-1681-z

**Published:** 2015-11-21

**Authors:** Alba Zhori, Marjol Meco, Helmut Brandl, Reinhard Bachofen

**Affiliations:** Department of Biology, University of Tirana, Tirana, Albania; Institute of Evolutionary Biology and Environmental Sciences, University of Zürich, Zurich, Switzerland; Institute of Plant Biology, University of Zürich, Zollikerstr. 107, 8008 Zurich, Switzerland

**Keywords:** *Euphorbia cyparissias*, *Uromyces* infection, Chlorophyll, Fluorescence transients, Variable fluorescence, F_v_/F_m_

## Abstract

**Background:**

Photosynthesis is the key process for plant growth and development. The determination of chlorophyll fluorescence kinetics allows the quantification of effects on photosynthetic processes triggered by environmental stress factors such as, e.g., the infection by fungal phytopathogens. The technique is non-invasive, rapid and well suited for experimental field work.

**Results:**

Healthy and *Uromyces*-infected plants of *Euphorbia cyparissias* were monitored directly in situ in the field using rapid fluorescence kinetics. Non-infected healthy plants show a typical maximum value for the relative variable fluorescence F_v_/F_m_ of around 0.8 with occasional variation between the leaves from the plant top towards the base, while infected plants exhibited a strong gradient to low values at the base. The photosynthetic performance index (PI) showed a higher heterogeneity within the leaves in both plant types.

**Conclusions:**

The non-invasive and rapid measurement of chlorophyll fluorescence induction allows characterizing the photosynthetic capacity of healthy and infected plants and of parts of them directly in the field. The PI, is highly sensitive not only concerning infection, but also towards other local environmental influences.

## Background

Photosynthesis is the basic process by which solar energy enters biological systems. Its efficiency is governed by many environmental parameters, such as light, temperature or nutrient availability. Special stress conditions may arise from infection by pathogenic organisms, fungi, bacteria or viruses. These may damage parts of the photosynthetic apparatus and decrease the photosynthetic efficiency of the infected plant.

*Euphorbia cyparissias* is widespread in middle Europe and found not only in the lowland, but also in alpine meadows up to 2600 m a.s.l. as a pioneer plant on dry and low nutrient soil [1–3]. It is perennial with vegetative reproduction by rhizomes and forms reduced flowers assembled in cyathia [4]. Quite frequently it is also known as host for the rust fungus *Uromyces pisi.* When *E. cyparissias* becomes infected, its morphology is strongly modified [5]. The flowers are lacking and the normal fine leaves are replaced by smaller thick oval shaped leaves, which are covered on their lower side with black spermogonia. After infection the leaves turn yellow from the leaf tip to the base and also a degeneration gradient concerning leaf size and morphology is observed along the stem from the vegetation point downwards to the roots. Early observations about infected plants by Tischler [6] indicate that new leaves outgrowing from the vegetation point of infected plants are healthy and not visibly infected by the fungus. Differences in the concentration of free sugar indicate physiological changes in the leaves which are correlated with the presence of the fungus. Alterations in plant hormone concentrations evidenced different changes in the physiology of the leaves already 60 years ago [7], but so far no information has been gained about perturbations in the energy metabolism of infected *Euphorbia* plants, especially regarding the primary processes of photosynthesis.

In other host-parasite systems photosynthesis becomes reduced after fungal infection, often parallel to a decrease in active leaf area and a loss in chlorophyll. Dark respiration is usually increased while transpiration seems variably influenced (e.g. as described by Bassanezi et al. [8] for three fungal pathogens).

**Fig. 1:**
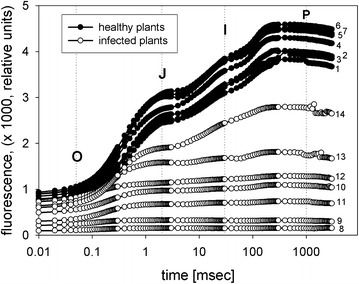
Fluorescence induction OJIP transient curves from leaves of healthy (*1*–*7*) and of infected *Euphorbia* *cyparissias* (*8*–*14*). Measurements are ordered from the *base* (*1* and *8*) to the *top* (*7* and *14*) of the plants

In the past decades chlorophyll fluorescence techniques have been used to follow primary processes in photosynthesis [9, 10] and since about 15 years they have been established to measure the viability of plants or the effects of stress upon plants due to environmental factors such as nutrition, drought, effects of air pollution or the effect of parasitic organisms. These techniques are non-invasive and rapid and ideal in field work, as reviewed two decades ago by Krause and Weis [11]. The resolution of the Kautsky transient [12] with high time resolution and log display results in a polyphasic rise of the fluorescence signal. This so called OJIP rapid kinetic test upon saturated illumination reveals detailed information not only on the redox state of the primary acceptor Q, but also on the electron transport beyond Q_A_ and on the donor side of photosystem II (PSII), and better allows to estimate the vitality of plants. In this test the fluorescence levels at different time steps between 10 µs (O = F_o_) and 1–3 s (P = F_m_) (see Fig. 1) are used to calculate the electron flow around PSII. The widely used F_v_/F_m_ value, equal to (F_m−_F_o_)/F_m_, gives the maximum quantum yield of PSII, where F_v_ is the variable fluorescence, F_m_ the maximum fluorescence at about 0.3 s and F_o_ the minimum fluorescence at the onset of illumination. However, this term covers only the first step of photochemistry in PSII [13]. It is therefore rather insensitive to detect metabolic changes beyond the primary photochemistry in the plant induced by environmental stress. By including the signal between step J and P information is gained on subsequent dark reactions of the photosynthetic electron transport beyond Q to photosystem I, allowing to calculate the photosynthetic performance index (PI), which translates the measured signals into parameters that allow to quantify the energy flow around PSII and beyond:

$${\text{PI}}_{\text{ABS}} = \left( {{\text{RC}}/{\text{ABS}}} \right)[\upphi_{\text{Po}} /\left( { 1- \upphi_{\text{Po}} } \right)\left] \, \right[\Psi_{\text{o}} /\left( { 1- \Psi_{\text{o}} } \right)],$$

where φ_Po_ is the maximum quantum yield of primary photochemistry, (F_m_ − F_o_)/F_m_, and Ψ_o_ is the probability that a trapped exciton moves an electron further than Q_A_^−^, = 1 − F_V2ms_. (RC/ABS) is the fraction of active reaction centers of PSII relative to the total light absorbing chlorophyll. Detailed information on the model and its theoretical basis is given in [14–16].

In the present study we describe the effects of *U. pisi* infection on the early events of photosynthesis in *E. cyparissias* by analyzing the fast chlorophyll fluorescence kinetics in the primary photosynthetic processes in the leaves on site in the field.

## Results

Time resolved fluorescence induction kinetics from healthy plants show the sequence of the steps of the reduction of the primary acceptor of PSII and the succeeding redox components in the electron transport chain between PSII and PSI in the OJIP test. Thus the fluorescence transients OJIP allow obtaining information on the site of action of the *Uromyces* infection.

Figure 1 gives a set of measurements from leaves of a healthy and an *Uromyces* infected *Euphorbia* plant, measured from the base of the plant to its top. F_o_ as well as F_m_ in both plants are increasing from base to top suggesting that the chlorophyll concentration in the leaves is increasing upwards. Both parameters may be used as approximation to the chlorophyll concentration in field experiments (11). While in healthy plants the typical three step transient kinetics OJIP with an increase in total fluorescence from the base to the top of the plant is seen, the maximum fluorescence in the infected plant is strongly diminished and only leaves near the top (leaf 13 and 14) demonstrate the normal three step transients.

**Fig. 2:**
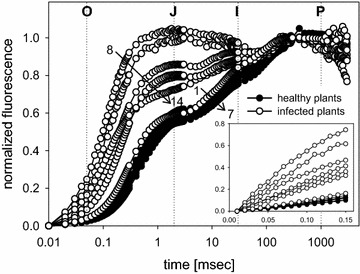
Relative variable fluorescence induction transients after double normalization between F_o_ = 0 and F_m_ = 1 for healthy (*1*–*7*) and infected *Euphorbia* *cyparissias* (*8*–*14*). Measurements are ordered from the *base* (*1* and *8*) to the *top* (*7* and *14*) of the plants. For normalization the value at time 10 µs was set 0 and the value at 0.3 s to 1 (data from Fig. 1)

When the transient fluorescence data are normalized by setting the values at 10 µs (F_o_) to zero and at 0.3 s (F_m_) to one, the seven traces of the healthy plant are found close together. From the base (1) to the top (7) a small time shift to the right is observed indicating a delay to reach the levels J, I and P. In infected plants the initial rise to the J-level is more rapid with only the exception for the top leaves (14) which come close to the healthy traces (Fig. 2). This is also seen in the initial rise kinetics (insert in Fig. 2). The mean value for healthy plants in the first 0.1 ms results in a slope of 0.82 ± 0.1 (normalized fluorescence units/msec) with minor variation, for infected plants the slope varies between 1.10 for the top leaf (14) and 4.98 and 6.11 for the heavily infected base leaves (8 and 9), respectively.

**Fig. 3:**
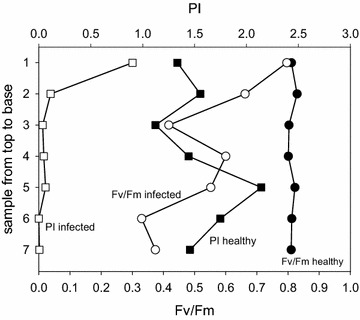
Profiles of the F_v_/F_m_ and PI values for healthy and infected *Euphorbia* plants. Measurements are ordered from the *top* (sample *1*) to the *base* (sample *7*) of the plants (data from Fig. 1). *Closed symbols* healthy plants, *open symbols* infected plants, *circles* F_v_/F_m_, *squares* PI

Most studies on the effect of environmental stress on plants use the ratio F_v_/F_m_ as indicator of plant vitality. It is not astonishing that the metabolic differences between healthy and infected *Euphorbia* plants are also clearly visible in the F_v_/F_m_ value (Fig. 3). In healthy plants F_v_/F_m_ is close to 0.8, a value many times documented in the literature for growing vegetation at non-stress conditions. In contrast, only the top leaves of infected plants may attain this value while in the lowest leaves often less than 0.4 is observed. In most experiments the F_v_/F_m_ value in infected plants stayed between 0.25 and 0.65, and in the top leaves it only occasionally reached the values of healthy plants (see Table 1). In contrast, the PI dropped in infected plants from 0.9 in the top leaves to values around 0.05 in leaf 3 and below it. In healthy plants the PI demonstrated a great variation which must be due to local differences in environmental stress factors.

**Table 1:** Average values of F_v_/F_m_ and PI values for healthy and infected leaves (no of samples)

	F_v_/F_m_	PI
Healthy, top of plant (14)	0.80 ± 0.03	2.29 ± 1.33
Healthy, base of plant (7)	0.67 ± 0.21	0.91 ± 0.85
Infected, top of plant (17)	0.69 ± 0.13	0.71 ± 0.84
Infected, base of plant (21)	0.51 ± 0.16	0.15 ± 0.21

T test showed that all F_v_/F_m_ and PI values, respectively, are statistically significant at P < 0.04

**Fig. 4:**
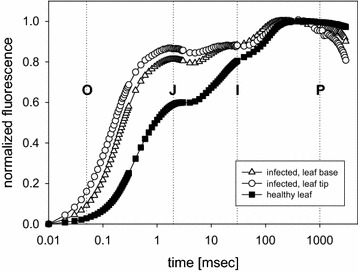
Normalized fluorescence induction transients (mean values of 5 measurements) from the leaf base and the leaf tip of infected *Euphorbia* plants, compared to healthy leaves. For double normalization the value at time 10 µs was set 0 and the value at 0.3 s to 1

The leaves of infected plants were not uniformly pigmented, the chlorophyll concentration shows visibly a gradient, suggesting that the pigment concentration is decreasing from the leaf base to the tip. The absolute fluorescence emission signal was up to five times larger for the leaf base compared to its tip (not shown). A difference between base and tip is also seen in the normalized transient curves, especially when both are compared with the signal from healthy leaves (Fig. 4). In the leaf tip the J-level is reached more rapidly compared to the base, indicating together with the reduced absolute signal size a stronger stress effect at the tip. Both transients show a faster rise of the fluorescence in relation to the uninfected control. The corresponding mean values of the F_v_/F_m_ drop from 0.69 for the leaf base to 0.35 for the leaf tip. In the evenly green leaves of healthy plants the differences in the induction transients between the base and the tip is small and the corresponding F_v_/F_m_ values stay close around 0.8 (Table 1).

In Table 1 the experimental values of the F_v_/F_m_ and of the PI are summarized. The young leaves of healthy plants vary little around 0.8 for the F_v_/F_m_, while the variability of the PI with a mean value of 2.29 covers a broad span from 1.1–6.1. The older leaves at the base show a decreased photosynthetic capacity with a mean F_v_/F_m_ of 0.67 and range between 0.25 and 0.82, while the PI (mean value 0.91) stayed within 0.3 and 2.1. Top leaves in infected plants realized F_v_/F_m_ values between 0.42 and 0.83 with a mean of 0.69, while the PI with a mean of 0.71 ranged between 0.01 and 2.9. At the base of infected plants the F_v_/F_m_ amounted for 0.21–0.74 (mean 0.51) and the corresponding PI was in the range between 0.01 and 0.73 (mean 0.15).

As stems of *Euphorbia* are green, they support plant photosynthesis. However, in uninfected plants the F_v_/F_m_ reached only 0.69 and the corresponding PI scored up to 0.85, both values in the range of the bottom leaves of healthy plants.

## Discussion

The data presented demonstrate that fluorescence induction kinetics is a rapid non-invasive tool to estimate the stress situation acting on plants and their organs. At the alpine sampling site at 2000 m a.s.l. many physical environmental factors are stress factors and may influence plant metabolism, such the rapidly varying temperatures and light conditions, the water loss by wind effects or the low nutrient and water availability due to heterogeneous soil structure. An infestation by parasites, in this case by the fungus *U. pisi*, may then intensify the stress situation of the plant. Although the samples have been collected within a range of few meters, all these stress factors may differ between the samples and the various measurements are not real replicates.

**Fig. 5:**
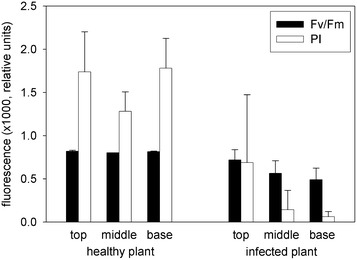
Normalized fluorescence induction parameters F_v_/F_m_ and the performance index PI for healthy and infected *Euphorbia* leaves

In many leaves of healthy plants the quantum yield of PSII, F_v_/F_m_, is mainly around 0.8, a value generally accepted for non-stress conditions. The PI however, covering also the electron transport towards photosystem I, shows a much greater variation. Leaves in the lower part of the plants near the soil often visibly tend to chlorosis, there the F_v_/F_m_ and PI values are lower and the variation between leaves is greatly increased (Fig. 5; Table 1). A clearly larger variation in both parameters is present in infected plants, with a strong gradient from the top to the base of the plants, only occasionally showing values of up to 0.8 for the top leaf. Still, the PI of these leaves is much lower compared to healthy plants with the same F_v_/F_m_, confirming the few previous investigations where F_v_/F_m_ and PI had been measured in parallel. Such variations are also well known for various trees after ozone treatment [17, 18].

The present observations on *Euphorbia* are in accordance with studies of a variety of infected plants where lowered F_v_/F_m_ values upon plant infection were observed. In beans (*Phaseolus vulgaris*) the quantum yield of PSII, F_v_/F_m_, dropped to zero with increasing infection time with *Phaeoisariopsis griseola* and *Colletotrichumlindemuthianum*; however, in contrast no effect was observed with *Uromyces appendiculatus,* independent on the grade of the disease [8]. In leaves of *Lupinus albus* infected with *Pleiochaeta setosa*, F_v_/F_m_ dropped to 0.4 [19]. Soybean rust (*Phakosporapachyrhizi*) on soybean (*Glycine max*) caused yield losses of up to 80 % parallel to a slight drop of F_v_/F_m_ from 0.82 to 0.7 at 50 % disease severity [20]. Infections of wheat leaves with Mildew (*Blumeriagraminis*) or rust (*Puccinia recondite*) had a strong effect on F_m_ and F_o_ [21] and fluorescence data correlated well with the severity of Cassava mosaic disease and the biomass yield per plant [22]. Fluorescence induction kinetics allows detecting the Esca disease in vine (*Vitis vinifera*) up to 2 months before morphological changes as foliar symptoms appear [23]. *Plantago ovata* is strongly affected by downy mildew (*Peronospora plantaginis*). Parallel to yield losses and increasing pigment chlorosis the F_v_/F_m_ ratio dropped from 0.82 for healthy, to 0.63 for slightly infected and to 0.47 for highly infected plants [24].

While the F_v_/F_m_ ratio is a normalized value between zero and one, and with a maximum around 0.8 for healthy plants, the PI for plants at good environmental conditions in contrast varies widely, as more factors affect it. So far no algorithm has been developed to calibrate the PI with carbon assimilation or biomass increase. However, a linear correlation between the PI and the plant length has been observed [25]. To quantitatively correlate the effect of a stress factor with the PI, the observed values are related to a corresponding control, a prerequisite usually not available in field experiments. Water stress resulted in a drop of the PI up to more than 80 %, depending on the sensitivity of the species of *Graptophyllum* [26]. Grapevine trunk disease reduced the PI by up to 10 %, similar to water stress [23]. In *Alternantheraphiloxeroides* treated with the product of the fungus *Nymbia alternantherae*, vulcolic acid, the PI sank by 70 % after 12 h of treatment, while the F_v_/F_m_ ratio dropped only by about 20 % [27]. This difference in sensitivity on an environmental effect is also seen in *Euphorbia* (Table 1; Fig. 5).

The fast fluorescence OJIP kinetics allows correlating physical reactions to structural organization in the PSII. The J-level covers the electron transport between the water splitting and the primary acceptor Q_A_. The high value of 0.8–1.0 for the J-level indicates a block at the site of the primary acceptor of PSII in infected plants. Q_A_ is reduced in a single turnover event, therefore in contrast to the healthy plants, the maximum fluorescence in infected plants is already reached within around 3 ms, the signal then declining towards the end of the measurement after 0.3 s.

**Fig. 6:**
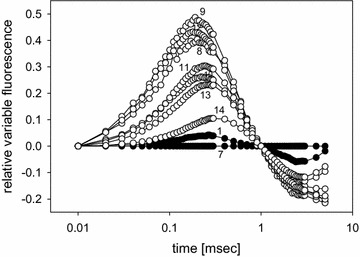
Differences between normalized fluorescence induction transients for healthy (*1*–*6*) and infected (*8*–*14*) *Euphorbia* leaves compared with the transient of the top healthy leaf (*7*). Normalization to 0 at 10 µs and 1 ms to demonstrate the K-level

Kinetic differences between the O and the J level indicate also limitations at the donor side of PSII at the K level at 0.2 ms [23, 25, 28]. The oxygen evolving complex is the most sensitive part of the photosynthetic electron transport towards physical stress like temperature and excess light. A normalization of the early part of the induction kinetics between 10 µs and 1 ms visualizes differences in the electron supply to PSII for healthy and infected plants (Fig. 6). It must be noted that the sequence in original fluorescence intensity (Fig. 1) and the normalized values (Fig. 2) from leaf 8 to 14 is not exactly transferred to the sequence of the differences to the kinetics of healthy leaves (leaves 1 and 7 in Fig. 6).

## Conclusion

Monitoring the vitality of a plant by time resolved fluorescence transients is a valuable tool to quantify site specific environmental stress factors. With handheld instruments measurements can be obtained on site and non-invasive with high spatial and temporal resolution. In general the technique allows quantifying stress conditions in space and time and describe the intensity of the development of parasitic infections from an early state on. The PI proofed to be much more sensitive than the more popular ratio of F_v_/F_m_.

## Methods

### Plant material

**Fig. 7:**
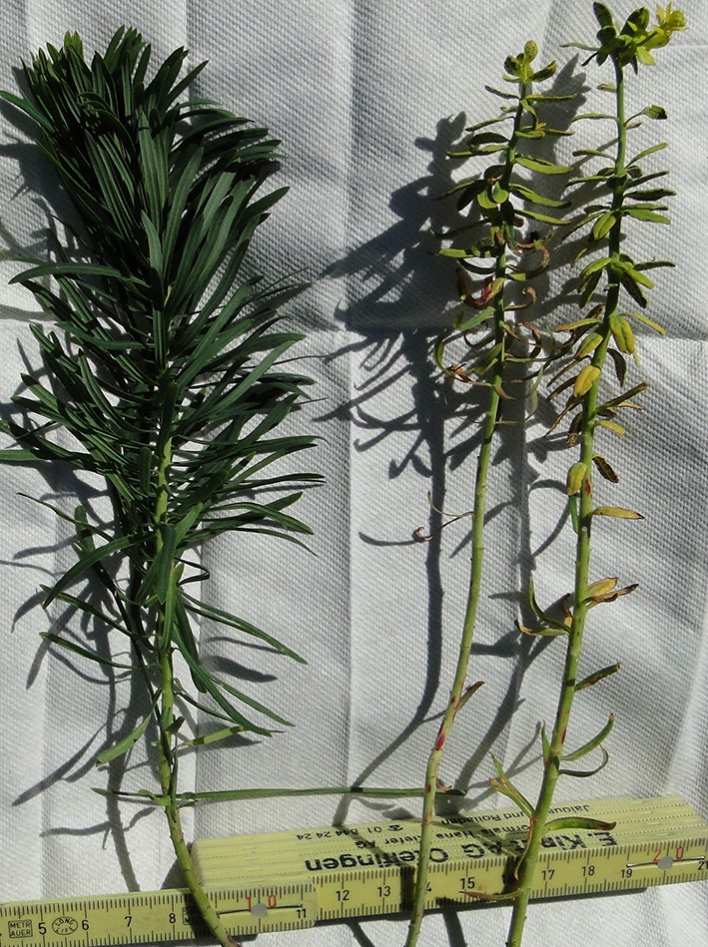
Healthy (*left*) and *Uromyces*-infected plant of *Euphorbia cyparissias*

Plants of *E. cyparissias* infected by *U. pisi* are frequently found. In the Piora valley infected and non-infected plants grow in mixed groups; infected plants amounted for between 10 and 50 % (Fig. 7). They have been sampled a few 100 m east of the Capanna Cadagno (46° 32′ 47.5′ north, 8° 43′ 10.8′ east) and analyzed immediately on the site.

### Chlorophyll fluorescence

Chlorophyll fluorescence was analysed using the portable fluorometer PEA (Hansatech, England). If possible the leaves were hold in commercial leaf clips (Hansatech, England) at the plant, leaf clips allow to keep the samples in the dark prior to the measurement (30 min) [14, 29]. For high spatial resolution leaves were detached and fixed in leaf clips. Fluorescence induction kinetics is measured with high temporal resolution during a saturation pulse. Excitation intensity was 3500 µmol photons m^−2^ s^−1^ with red light of 650 nm for 3 s. From the fluorescence induction signal from 10 µs to 3 s the instrument determines initial (F_o_) and maximum (F_m_) fluorescence and the variable fluorescence (F_v_) at specified time intervals. Furthermore it calculates specific parameters such as the potential quantum yield of PSII (F_v_/F_m_), the performance index (PI), the area between F_o_ and F_m_ or the time to reach F_m_. The PI includes besides the F_v_/F_m_ also the ratio of active reaction centres compared to the chlorophyll, as well as the dark redox reactions beyond the acceptor system of PSII. Detailed information on the theory behind the PI is discussed in [14–16]. For calculations the experimental fluorescence data were exported into Excel.

## Abbreviations

PI: photosynthetic performance index
PSII: photosystem II
